# Incorporating Person Centred Care Principles into an Ongoing Comprehensive Cancer Management Program: An Experiential Account

**DOI:** 10.4103/0973-1075.76245

**Published:** 2011-01

**Authors:** Vallath Nandini, CN Sridhar, MR Usharani, John Preshanth Kumar, Naveen Salins

**Affiliations:** Consultant and Coordinator, Department of Integrative Oncology, HCG- Bangalore Institute of Oncology, Speciality Centre, Bangalore, India

**Keywords:** Integrative oncology, Multi-disciplinary team, Palliative, Quality of life

## Abstract

Recent research indicates a definite positive impact on treatment outcomes when an integrative approach that focuses on symptom control and quality of life is provided along with the standard therapeutic regimens. However implementation or practice of this approach is not seen widely due to the culture of medical training and practice. This article presents the initial development of a program for incorporating integrative care principles into an ongoing comprehensive cancer care program at a tertiary centre. The key purpose of the program being to develop, facilitate, and establish comprehensive and holistic processes including palliative care principles, that would positively enhance the quantity and quality of life of the person with disease, as well as create an environment that reflects and sustains this approach. The vision, objectives, goals, strategies, activities and results within the 7 months of implementation are documented. The new learnings gained during the process have also been noted in the hope that the model described may be used to conceptualize similar care giving facilities in other centres.

## INTRODUCTION

Recent research indicates a definite positive impact on treatment outcomes when an integrative approach that focuses on symptom control and quality of life is provided along with the standard therapeutic regimens.[1–6] However implementation or practice of this approach is not seen widely due to the culture of medical training and practice.

In our country, there is wide demarcation between disease modifying therapies for cancer and integrative and palliative care efforts. The specialists in palliative care often work in islands; be it as stand-alone outpatient / in patient service, home based or hospice services.

This article presents the initial development of a program for incorporating integrative care principles into an ongoing comprehensive cancer care program at a tertiary centre.

## BACKGROUND

In a typical tertiary cancer care institution, as illustrated by the institute in which this program was initiated, the needs of care in cancer patients are perceived predominantly as disease modifying interventions. For managing the significant symptom burden of cancer patients[7,8] there usually was a system in place to provide some symptom relief for certain patients with advanced disease through the expertise of oncologists, anesthesiologists and a visiting part time palliative care specialist.

### The integrative oncology program

The program envisioned a department that embodies a comprehensive and sustainable model of holistic care for persons affected with cancer that enhances well being at the physical, emotional, social and spiritual dimension. The key purpose of the program being to develop, facilitate, and establish comprehensive and holistic processes including palliative care principles, that would positively enhance the quantity and quality of life of the person with disease, as well as create an environment that reflects and sustains this approach.

### The integrative oncology program objectives

1. Holistically assess the concerns of the person who has cancer and incorporate patient centred care to the ongoing cancer care program[9]
2. Seek and provide appropriate multidisciplinary inputs from the team of experienced professionals
3. Assure competent, compassionate, and continued care through out
4. Enhance the experience by nurturing factors that improve quality of life
5. Systematically record and study the processes in place and provide appropriate inputs to modify the culture and enhance the quality of care provided and perceived.

### Goals

1. For clinical care
  - Referrals for pain, symptom management and supportive care from few motivated oncologists within the 1^st^ month
  - Referrals for pain and symptom management and supportive care from 50% of consultants within 12 months
  - Integration of appropriate measures for pain, symptom management and supportive care within the oncology care protocols within 2 years
  - Implementation of appropriate measures for pain, symptom management and supportive care according to the prescribed care protocols over years
2. Working with the multidisciplinary team
  - Regular networking with the existing systems of care within the hospital set up aimed to integrate and enhance the quality of care[10]
  - Regular focused interaction on specific aspects of the quality of care, within the group thus formed
  - Formulation of specific care protocols from the inputs
3. Formation of a department of integrative oncology
  - With multidisciplinary team members including motivated consultants from different streams, paramedical professionals, quality cell/ administration representation and patient advocates
  - Incorporation of additional medical and nursing staff trained and experienced in palliative care to enhance the capacity of the team.

### Operational strategies

1. Medical
  - Identify and work together with a few oncologists who show inclination towards the concept
  - Keep the primary team involved with the process of care for as much and for as long as feasible even beyond the curative stage.
  - Inspire interest amongst professionals in general through
    - informative presentations based on *data derived from within the hospital* patients
    - regular evidence based academic presentations
    - invited lectures from authoritative personalities from different backgrounds e.g. medical specialities including the field of supportive care and oncology, law, sociology, physical medicine and rehabilitation, complementary and alternative medicine etc
2. Nursing
  - Build skills to improve quality of care through targeted training activities incorporated into the ongoing oncological nurse training program.
  - Build general palliative nursing skills and attitudes - to result in overall capacity building. Do not to recruit a specialist palliative care nurse in the beginning; a specialist nurse could be inducted when the program is well entrenched and has significant volume.
3. Process
  - Focus on a slow culture change through continued informed interactions at individual and group levels.
  - Work closely with the hospital *quality control cell* to influence policy and compliance.
  - Concentrate on the process and not get distracted by individual case based incidents, decisions, or outcomes that may appear divergent from care principles.

### Areas of activities to assure sustainability

Picture- Figure 1a and b

**Figure 1a:**
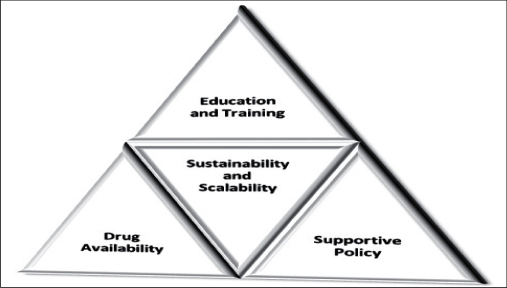
Domains of action for integration

**Figure 1b:**
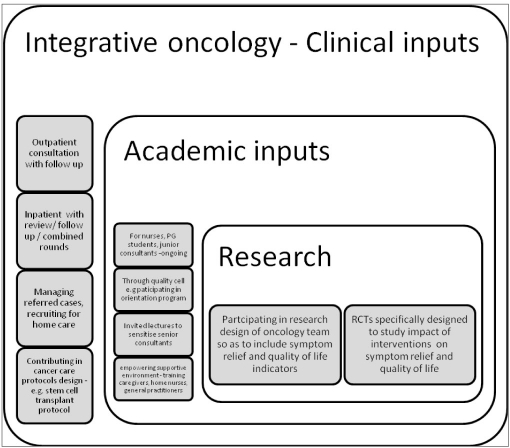
Integrative Oncology inputs within the system

### Influencing policy

1. As pain relief is a perceivable outcome appreciated by all care givers. “Pain relief Needs Assessment” survey was presented to policy makers as
  - Statistics of prevalence of pain amongst IP patients
  - Listing of gaps, in the process of care, from acknowledging the presence of pain up-to the point of analgesic interventions reaching the patient and documentation of pain relief
  - Presenting specific measures for gap closure, including the training needs
2. Pain policy and end of life care policy document was created working closely with the Quality Control Cell of the hospital [NABH (National Accreditation Board for Hospitals) accreditation]. This provided the impetus and space for documentation of pain score, related teaching programs, patient awareness programs and alterations of prescription patterns along with monitoring of all the realms.

### Facilitating availability of pertinent drugs to encompass symptom control

1. Regular interactions with oncologists through combined rounds and focused discussion of symptoms to influence prescription patterns
2. Process of updating the pharmacy stores is ongoing with the list of essential drugs based on WHO recommendations
3. The renewal of license for oral Morphine with defined prescription rights
4. Information on accessing liquid oral morphine in the area made available for needy patients

### Education and training

Doctors were often willing to help; less open to getting trained where as nurses were willing to get trained but often constrained, due to the existing hierarchical systems at personal and professional levels.

There was the favourable climate within the centre as it was undergoing the process of NABH accreditation. Hospital policies and support of the administration and quality control cell helped in initiating various academic activities

1. The IAPC (Indian Association of Palliative Care) certificate course was utilized to enroll nurses and junior consultants into the concepts. Its advantages were brevity, nationwide acceptability, link up with opioid license, and availability of national faculty.
2. Specific topics that further emphasizes and clarifies the concepts are being incorporated, within the ongoing the academic program of the hospital E.g.
3. Practical aspects of using strong opioids
4. Impact of psychosocial dimensions of care on treatment outcomes in cancer patients
5. Colostomy care, Lymphedema care
6. Prognostication and transition of care
7. Decision making based on ethics in situational dilemmas
8. Shared decisions as empowered autonomy
9. Workshops on communication skills
10. Our strategies for keeping the consultants in loop during the process of individual patient care were mainly in the following domains
11. Involving consultants for additional input during assessment of patients and understanding of symptoms with progression of disease e.g. opinion on palliative radiotherapy
12. Discussing interesting clinical details e.g. excessive sweating on half of the face in a patient with progressive apical lung cancer
13. Maintaining review of patients with the primary consultants after symptoms are controlled.
14. Conveying information through relatives regarding death and the support perceived during all the stages including the terminal phase.

### Initiating a supportive social environment for continued care

1. Education and training of family members to continue and enhance patient care
2. Networking with general practitioners in the patient’s neighbourhood for continued clinical care
3. Assisting in getting in touch with trained home nursing teams and having a transition training period in the IP setting
4. Linking up with other ongoing home based hospice care programs in the neighbourhood
5. A Proposal (waiting for approval) for home based care for patients registered with the institution
6. Conducting regular workshops and seminars on the basic concepts and practical aspects of caring for persons with long term illnesses in regional hospital and community settings
7. Active advocacy by bringing together and working in partnership with representatives of government and other NGO (Non Government Organisation) s in the state with the aim of
  - Influencing *policies to increase access and capacity* for whole person based care at state and national levels
  - Initiate training programs for medical and nursing professionals in various urban and rural settings
  - Mobilize community
  - to increase awareness and demand for care
  - to influence channelization of social capital
  - to inspire community ownership and empowerment for sustainability of care programs

## RESULTS

### Clinical care

**Figure 2:**
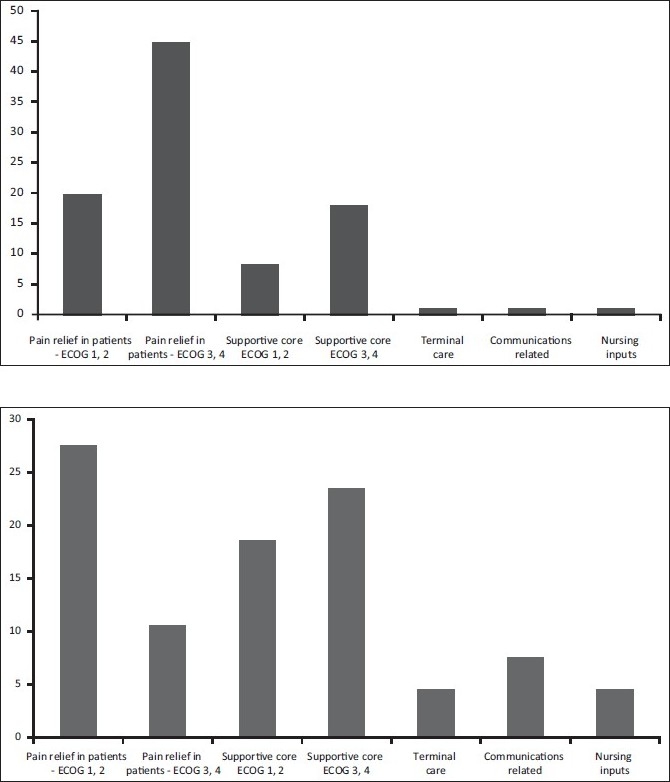
Changes in the pattern of references

1. Changes in pattern in referrals of patients at different stages of diseases expanded from pain management to symptom relief at different stages, nursing care and communication related references - Figure 2
2. Access to integrative care is helping patients to go through disease modifying therapy with lesser distress and reduced symptom load. [analytical data being compiled]

### Policy formation

1. Ongoing implementation of pain relief policy
  - Modification of the hospital vital chart document to include pain scale as the 5^th^ vital parameter - [Figure- 3a and b] Comparison of pain scores at different intervals during the program
  - Design and display of posters on WHO (World Health Organisation) analgesic Ladder in outpatient / in patient settings, list of drugs in each step with dose and frequency
  - Regular classes on different aspects of pain management
  - Patient empowerment through information on available services
2. End of life Care document has been formulated. Efforts are ongoing to establish it as part of continued care through appropriate documentation and feed back to the concerned oncology team. This has been implemented in a few cases of patients who had been specifically referred for the same.
3. Patient information booklets are being prepared, with specific information on disease treatment and management e.g. handling fatigue, insomnia etc, beneficiary diet inputs, early detection and prevention of complications, etc.

**Figure 3a:**
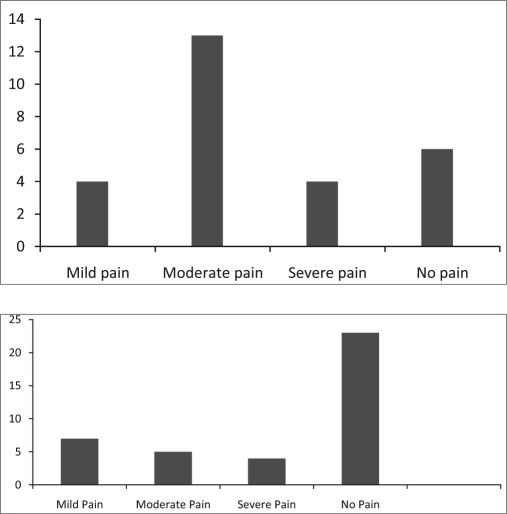
Comparison of distribution of pain scores before and after instituting the program

**Figure 3b:**
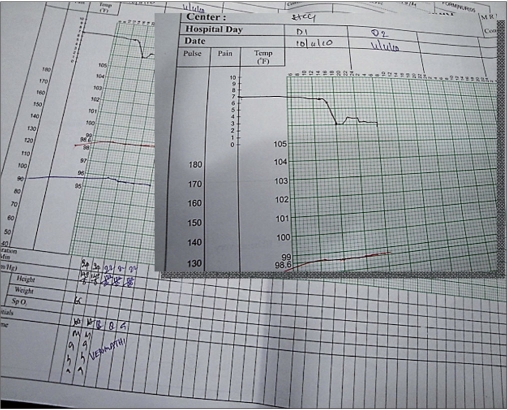
Pain Score as the 5^th^ Vital parameter

### Drug availability and usage

**Figure 4:**
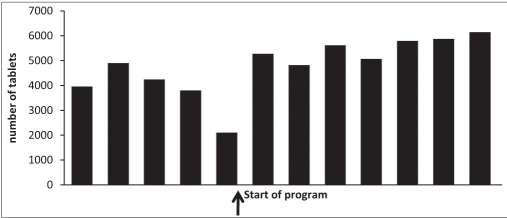
Oral Morphine utilisation

1. Ensured availability of symptom control drugs in the pharmacy and emergency drugs kit in the ward settings
2. Observed uptick in the utilization of analgesics - Figure 4

### At the process level

#### Institution

Establishment of the “Department of Integrative Oncology” with multidisciplinary team members with similar goals related to improving the quality of life of the “person who has the disease”

1. Medical professionals
  - Palliative Medicine specialists
  - Oncologists, anaesthesiologists, physician, speciality surgeons, psychiatrist
2. Nursing professionals
  - Oncology nurses who understand and practice the concepts of palliative care nursing
  - Specialist palliative nurses
3. Clinical pharmacologist, Dietitians, Physiotherapists, Psychologists, Medical Social Worker
4. Specialists in Complementary and Alternative Medicine
5. Administrator representative

#### Community

1. Home based care for individual patient as needed with the above networking.
2. A successful workshop with the participation of the major NGOs in the state and authorities in the field, has conceived the blue print for a systematic approach to enhance capacity and access to care programs within the state. The nodal persons, centres and activities have been identified. The journey has begun in good faith.

### Attitudinal shifts represented by

1. Expansion in the type of references - Figure 2
2. Ongoing regular combined rounds with oncologists
3. Combined outpatient consultation in certain oncology settings
4. Working with the oncology team for incorporating symptom management and supportive care protocols within the individual cancer management protocols
5. Inclusion of speciality within new initiatives e.g. Bone Marrow transplant program
6. Combined research projects e.g. integrated approach to fatigue management in ambulatory cancer patients undergoing chemotherapy
7. Creation of web page for the department with its vision/ mission statements, objectives, activities and link to the MDT (Multi Disciplinary Team) inputs

### Learnings

#### Through interactions with the oncology team

The fundamental learning was the acknowledgement and understanding of the dynamics and equations between the patient and primary consultant and to add value to the quality of care working within this space. Given below are some of the germane learnings acquired within this space

**Figure 5:**
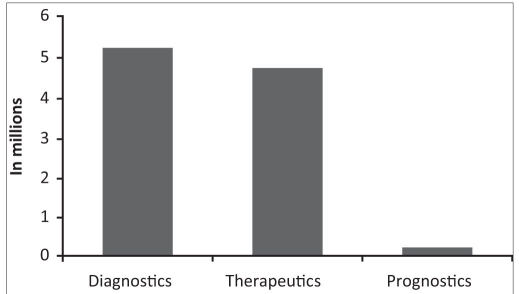
Number of publications in clinical domains

**Figure 6:**
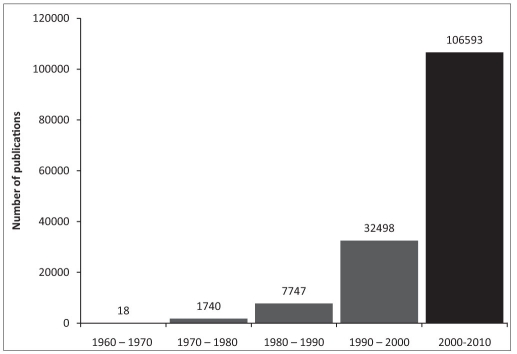
Pubmed search results for articles on quality of life

1. Better understanding of the barriers amongst oncologists to utilise palliative care knowledge base
  - Incomplete awareness about the scope of palliative medical and nursing inputs
  - Speciality of oncology is guided mainly by research. Most studies concentrate on disease modifying interventions.[11] The primary objectives of most oncological researches do not cover the impact of intervention under study on symptom control, pain relief or quality of life.[12] Thus symptoms may be perceived as of secondary importance, and as divergence from the main professional objective of disease modification.
  - Medical training and care systems are unsupportive of handling uncertainty while deciding clinical management. Prognostication being an under researched domain has not entered into the training or practice levels - Figure 5
  - Lack of clarity and training in utilising medical ethics and communication skills for empowered and shared decision making
  - Dearth of institutional policies related to life supporting interventions. The CPR (Cardiopulmonary resuscitation) and other intensive care training programs usually do not include the ethical aspects of when it would be a futile intervention and when it would not be in the best interest of the patient.
2. With regular interactions, and further relevant research, this scene would be modified e.g. Quality of life is beginning to show its presence as an important research objective - Figure 6
3. Learnings in clinical care
  - Interacting with the primary consultant during assessment / evaluation of a particular symptom is clearly a learning process that assures more competence
  - There are diagnostic and investigational advantages while working within a comprehensive cancer care program
  - Knowledge base gets deeper and wider e.g. - e.g. checking for neutropenia is important in patients undergoing chemotherapy before ordering a simple intervention like high up enema for constipation as this may lead to an infectious bout.
  - The relevance of correct choice of antibiotic and antifungal regimens based on the immune status of the patient.
  - Noting the high incidence of correctable micronutrient deficiencies e.g. Vitamins, magnesium
  - More rigor is elicited in assessing and treating symptoms by the palliative care specialist, as each decision is accountable to scrutiny.

#### Learnings related to integrating with multidisciplinary team

Contribution of each member adds value to the total care perceived by the patient

1. Prophylactic physiotherapy inputs for delaying and containing dyspnea in selected patients
2. The clinical pharmacologist’s presence makes prescription review/modification more proficient.
3. Incorporating dietary inputs in clinical situations curtails the total number of medicines that patient has to take - e.g. morphine induced constipation, radiation enteritis, anemia, fatigue, leucopenia, thrombocytopenia.
4. Use of alternative medical inputs are being found useful in certain symptom management protocols - e.g Ayurveda formulations in constipation[13] mucositis, yoga inputs[6] in anxiety neurosis, insomnia, fatigue and certain categories of pain

#### Learnings about the differences in Patient’s expectations in an integrated set up

1. The hope for cure is extremely strong. Hence even the slightest hint is taken as a high possibility.
2. There is an expectation to have significant communications directly from primary consultants
3. The family and patients expect detailed explanation and clarifications regarding interventions initiated or withheld

## CONCLUSIONS

A survey conducted by ESMO (European Society of Medical Oncology) amongst medical oncologists brought forth an interesting observation that the ‘practitioners in private practice or teaching hospitals had substantially more positive attitudes regarding palliative care compared with physicians based in comprehensive cancer centres (*P*- 0.05)[14] For improved access to appropriate care, there is a need for incorporating principles and concepts of integrative and palliative care within the culture of health care institutions and comprehensive cancer care programs.

As a long term vision, this integration needs to expand to all the settings where a need may be perceived i.e. hospitals in the government and non- government sectors and into the mind set of practicing medical / paramedical professionals and other comprehensive programs e.g. HIV care, dementia care etc.

The greatest beneficiary of integrative oncology principles into mainstream cancer management would be the patient, in terms of *quality as well as quantity of life*. The regular inputs from the MDT makes the therapeutic experience more acceptable to the patient and hence there are higher possibilities of completion and cure. It allows continuity of care, support, early assessment and management of symptoms and augmented physical well-being. It would also determine primacy and allow appropriate person-centred-goals of care at various stages of the disease.

Integration will in turn influence the rigor of establishing therapeutic regimens in Palliative Medical practice, make it more evidence based, and at the same time enhance access of patients under its cover to relevant curative therapy.[11]
